# Cryopreservation of tissues by slow-freezing using an emerging zwitterionic cryoprotectant

**DOI:** 10.1038/s41598-022-23913-3

**Published:** 2023-01-02

**Authors:** Takeru Ishizaki, Yasuto Takeuchi, Kojiro Ishibashi, Noriko Gotoh, Eishu Hirata, Kosuke Kuroda

**Affiliations:** 1 Faculty of Biological Science and Technology, Institute of Science and Engineering, Kanazawa University, Kakuma-Machi, Kanazawa, 920-1192 Japan; 2 Cancer Research Institute, Kanazawa University, Kakuma-Machi, Kanazawa, 920-1192 Japan; 3 WPI-Nano Life Science Institute, Kanazawa University, Kakuma-Machi, Kanazawa, 920-1192 Japan; 4 NanoMaterials Research Institute, Kanazawa University, Kakuma-Machi, Kanazawa, 920-1192 Japan

**Keywords:** Biomaterials - cells, Biomaterials - cells

## Abstract

Cryopreservation of tissues is a tough challenge. Cryopreservation is categorized into slow-freezing and vitrification, and vitrification has recently been recognized as a suitable method for tissue cryopreservation. On the contrary, some researchers have reported that slow-freezing also has potential for tissue cryopreservation. Although conventional cryoprotectants have been studied well, some novel ones may efficiently cryopreserve tissues via slow-freezing. In this study, we used aqueous solutions of an emerging cryoprotectant, an artificial zwitterion supplemented with a conventional cryoprotectant, dimethyl sulfoxide (DMSO), for cell spheroids. The zwitterion/DMSO aqueous solutions produced a better cryoprotective effect on cell spheroids, which are the smallest units of tissues, compared to that of a commercial cryoprotectant. Cryopreservation with the zwitterion/DMSO solutions not only exhibited better cell recovery but also maintained the functions of the spheroids effectively. The optimized composition of the solution was 10 wt% zwitterion, 15 wt% DMSO, and 75 wt% water. The zwitterion/DMSO solution gave a higher number of living cells for the cryopreservation of mouse tumor tissues than a commercial cryoprotectant. The zwitterion/DMSO solution was also able to cryopreserve human tumor tissue, a patient-derived xenograft.

## Introduction

Efficient long-term preservation of tissues is a tough challenge and novel technologies for the long-term preservation of various tissues are in high demand. Cryopreservation is a key technology for long-term preservation and has already been well established for single dispersed cells. Cells are generally cryopreserved in a deep freezer at around − 80 °C or in liquid nitrogen at − 196 °C. Cryoprotective agents (CPAs) have been developed to prevent physical damage to cells induced by ice crystals formed under extremely low temperature^1^. The most commonly used CPAs are glycerol^2^ and dimethyl sulfoxide (DMSO)^3^. Although some papers have reported cryopreservation of multicellular spheroids and tissues using these CPAs with a certain degree of cell viability^4–8^, generally, cryopreservation is inadequate for multicellular systems.

Cryopreservation is categorized into slow-freezing and vitrification^9^. Slow-freezing has a low cooling rate and prevents intracellular ice formation (IIF) by dehydration of cells. Slow-freezing is widely used for dispersed single cells because it works even with low concentrations of toxic CPAs and the amateur skills of the operator. Vitrification has a high cooling rate and prevents IIF by the instantaneous formation of a glass-like structure^10^. Although it requires high concentrations of toxic CPAs and expert skills, it has recently attracted attention because it completely avoids ice crystal formation. In addition, vitrification leads to low volume change at low temperatures, avoiding compression of the tissues. Based on these advantages, vitrification is recognized to be suitable for the cryopreservation of tissues; it is performed using polyvinylpyrrolidone^11–15^, polyvinyl alcohol, polyglycerol^13–15^, polyampholytes^16^, and conventional CPAs. Though slow-freezing of tissues has not, therefore, been enthusiastically studied in recent years, Lee et al*.* recently reported that slow-freezing is superior for cryopreservation of ovarian tissues^17^. We here considered that slow-freezing has untapped potential. In this study, the emerging CPAs were applied to harness this potential, because conventional CPAs have already been well studied.

**Figure 1:**
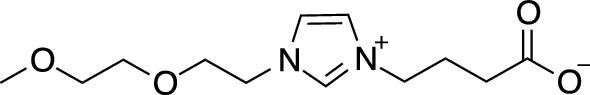
Chemical structure of the imidazolium/carboxylate zwitterion used in this study**.**

We have proposed low-molecular-weight aprotic synthetic zwitterions as novel CPAs for slow-freezing (Fig. 1)^18^. Zwitterions have positive and negative charges in one molecule. The synthetic zwitterions applicable to cryopreservation possess various cations such as imidazolium, ammonium, and pyridinium and anions such as carboxylate and sulfonate. They have low cytotoxicity. For example, the median effective concentrations (EC_50_) of zwitterions to *E. coli* are higher than DMSO, ethanol, and typical ionic liquids^19,20^. An imidazolium/carboxylate zwitterion gives higher viability of human normal fibroblasts than DMSO after 24 h of cultivation at 10% (v/v). The same tendency is obtained for zebrafish embryos^18^. The zwitterion did not kill zebrafish embryos at 5% but the same concentration of DMSO killed most of them. Zwitterions we synthesize are aprotic, and therefore they basically always possess electric charges unlike protic zwitterions such as amino acids. The constant electric charges strongly interact with water, resulting in the inhibition of ice crystallization^21^. Therefore, the zwitterions show a similar effect to that of commercial CPA for single cells. Although synthetic zwitterionic polymers^22,23^ and natural zwitterions^24–26^ as CPAs have been studied in detail, low-molecular-weight synthetic zwitterions have not been well studied.

The synthetic zwitterions are cell-impermeable CPAs and cannot inhibit IIF directly, unlike cell-permeable natural zwitterions^18,24–26^, however, they increase the osmolarity of the freezing medium and indirectly inhibit IIF by dehydrating cells due to the high osmotic pressure. Moreover, their cryoprotective effect is improved by the supplementation of cell-permeable DMSO. In a preliminary study, we demonstrated that some aqueous mixtures of zwitterion/DMSO showed a higher cryoprotective effect than a commercial CPA on a variety of single dispersed cells vulnerable to freezing^21^. In this study, the zwitterion/DMSO aqueous solutions were applied to the cryopreservation of tissues. Tumor tissues were used for this primitive study because of convenience. While there are many zwitterion species, we used the imidazolium/carboxylate zwitterion (called OE_2_imC_3_C in our previous works) shown in Fig. 1 because it is a promising species^21^.

## Results and discussion

### Cryopreservation of spheroids

**Figure 2:**
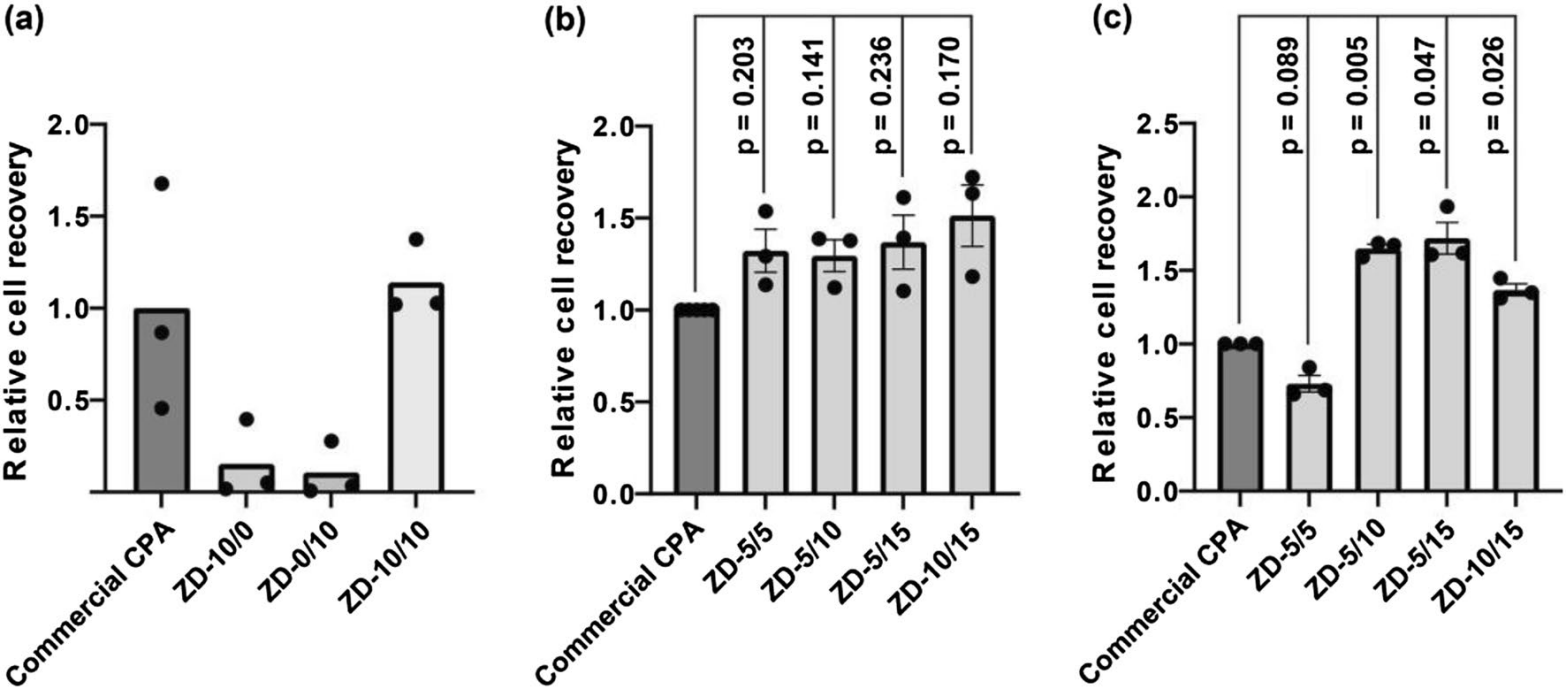
Relative cell recoveries of cryopreserved mouse melanoma cell spheroids post-thaw using different mixtures of zwitterion and DMSO. (**a**) Initial tests on non-optimized mixtures with cell recoveries measured immediately post-thaw (*n* = 1, experimental triplicates). (**b**) Optimization of mixture concentrations with cell recoveries measured immediately post-thaw (*n* = 3, biological triplicates). (**c**) Optimization of mixture concentrations with cell recoveries measured after 24 h of incubation post-thaw (*n* = 3, biological triplicates). The bars show standard errors. The commercial CPA employed is CultureSure® freezing medium (Fujifilm Wako Pure Chemical Corporation).

Spheroids, the smallest units and building blocks of artificial tissues, were cryopreserved. Cryopreservation of even small tissues such as spheroids is known to be challenging compared to that of single dispersed cells. Spheroids were created using mouse melanoma cells (5555). The relative cell recovery is normalized with values of a commercial CPA (CultureSure^®^ freezing medium, Fujifilm Wako Pure Chemical Corporation) containing DMSO and bovine serum albumin. When using zwitterion/DMSO/water (10/0/90, w/w/w: ZD-10/0), relative cell recovery after cryopreservation was 0.15 (Fig. 2a, Fig. S1), indicating ZD-10/0 was not effective. Zwitterion/DMSO/water (10/10/80, w/w/w: ZD-10/10), which is expected to have a higher cryoprotective effect, gave a relative cell recovery of 1.14 and thus was comparable to the commercial CPA. Zwitterion/DMSO/water (0/10/90, w/w/w: ZD-0/10) gave a relative cell recovery of only 0.11, indicating the importance of mixing the zwitterion and DMSO.

Cryopreservation of dispersed single 5555 cells resulted in higher cell recoveries (Fig. S2), compared to those of the spheroids. The relative cell recoveries of dispersed single cells were 0.71 and 0.46 for ZD-10/0 and ZD-0/10, respectively. Those for spheroid were 0.15 and 0.11, respectively. Therefore, the cryopreservation of multicellular systems was confirmed to be more difficult than that of single cells although cell concentration was different (for dispersed cells and spheroids: 1 × 10^7^ vs 8 × 10^5^ cells/mL). The zwitterion indirectly inhibits IIF by dehydrating cells. The dehydration gives sufficient cell recovery for the cryopreservation of single cells. However, IIF in any single cell of the multicellular systems is critical because the intracellular ice inflows into adjacent cells through the gap junctions^27^. Therefore, cryopreservation of multicellular systems only with the zwitterion is difficult, while the mixture with DMSO exhibits a synergistic cryoprotective effect. Combining cell-permeable and cell-impermeable CPAs for tissues is typical^28^, and ZD-10/10 is one following the typical tactic. Because the combination of zwitterion and DMSO is comparable to the commercial CPA despite the unoptimized composition, the specifically optimized composition is assumed to be promising.

Twelve solutions with different zwitterion/DMSO concentrations (ZD-5/5 to ZD-20/15) were prepared and optimized (Fig. S3a–c). ZD-5/5, ZD-5/10, ZD-5/15, and ZD-10/15 especially produced higher relative cell recoveries immediately post-thaw (1.32, 1.30, 1.37, and 1.51, respectively) (Fig. 2b, Fig. S4), compared to that of the commercial CPA. Although there is no statistical significance (*p* value ≥ 0.141), all the values were higher than those obtained with the commercial CPA. The cell viabilities and recoveries of spheroids, shown in Fig. S3, decreased when the concentration of the zwitterion was 15 wt% or more. This trend was also observed in the cryopreservation of single cells (Fig. S5). The zwitterion is cell-impermeable; therefore, cells may have been killed by the excessively high osmotic pressure.

The abovementioned results are based on the relative cell recoveries immediately after thawing, but, sometimes, “false-positive” recovery results are obtained where cells appear as viable immediately post-thaw, but apoptotic and necrotic activity may only translate into cell loss after some time of culture post-thaw^29,30^. To avoid false positives, we evaluated the relative cell recoveries of spheroids after 24 h of incubation from thawing, using our four optimized solutions (ZD-5/5, ZD-5/10, ZD-5/15, and ZD-10/15; Fig. 2c, Fig. S6). ZD-5/10, ZD-5/15, and ZD-10/15 produced high relative cell recoveries (1.65, 1.72, and 1.37, respectively). The absolute cell recoveries with ZD-5/10 and ZD-5/15 were almost 100% (Fig. S6). In the case of ZD-5/5, although the relative cell recovery immediately after thawing was 1.32, after 24 h, it decreased to 0.73. This is the case of “false positive”. The cell viability with ZD-5/5 after 24 h of incubation was still high (Fig. S6) despite the low cell recovery, indicating that the dead cells by apoptosis disintegrated into fragments during 24 h of incubation.

The ability for spheroids to naturally fuse when in close contact with each other is a feature that was investigated post-thaw to see if any of the optimized solutions altered this behavior compared to the commercial CPA and fresh spheroids (Fig. S7). The spheroids cryopreserved with all solutions used in this study, including the commercial CPA, fused within 5 days. Their behavior was similar to that of the fresh spheroids.

Melanoma-associated fibroblast (MAF1) spheroids were also cryopreserved (Fig. S8). All four optimized solutions showed higher cell recoveries and viabilities immediately post-thaw than the commercial CPA. This trend is similar to that observed with 5555 spheroids.

### Cryopreservation of 5555/MAF1 co-cultured spheroids

**Figure 3:**
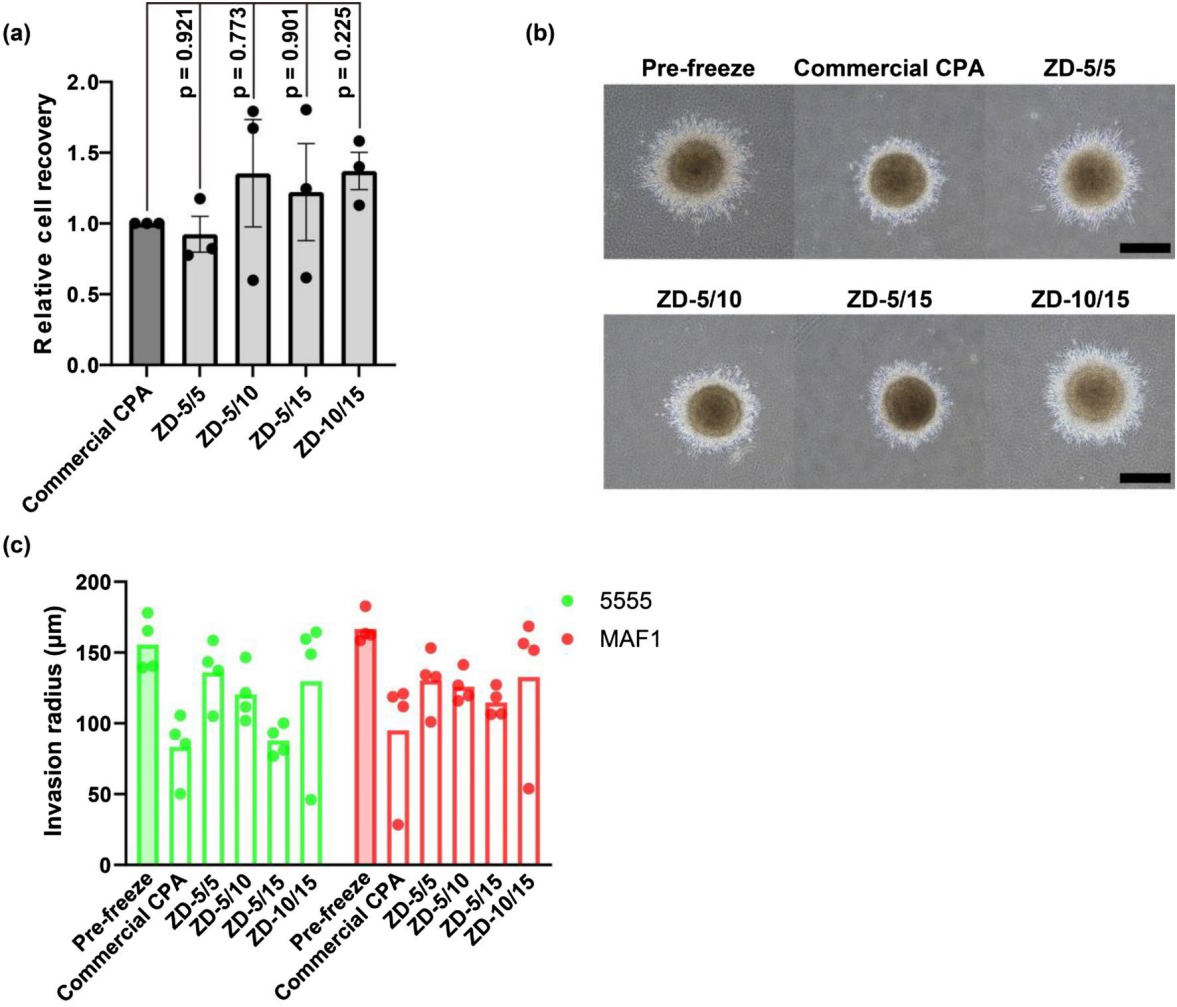
Relative cell recoveries and invasion ability of cryopreserved 5555/MAF1 co-cultured spheroids post-thaw using optimized mixtures of zwitterion and DMSO. (**a**) Relative cell recoveries of cryopreserved 5555/MAF1 co-cultured spheroids measured immediately post-thaw (*n* = 3, biological triplicates). (**b**) Typical images for invasion of spheroids after 24 h of incubation post-thaw. All images are in Fig. S11. Scale bar represents 500 µm. (**c**) Invasion radius of spheroids after 24 h of incubation post-thaw (*n* = 1, experimental quadruplicates). The bar shows standard errors. The commercial CPA employed was CultureSure® freezing medium (Fujifilm Wako Pure Chemical Corporation).

Co-cultured spheroids of 5555 and MAF1 were cryopreserved because natural tissues consist of multiple types of cells (Fig. 3a, Fig. S9). Again, the four optimized solutions were used. Relative cell recoveries immediately post-thaw with ZD-5/5, ZD-5/10, ZD-5/15, and ZD-10/15 were 0.92, 1.36, 1.22, and 1.37, respectively. ZD-10/15 exhibited the highest cryoprotective effect: all plots of ZD-10/15 were higher than those with the commercial CPA although the difference was not statistically significant (*p* values ≥ 0.225).

The fusion ability of 5555/MAF1 co-cultured spheroids after cryopreservation was also investigated (Fig. S10). Fresh spheroids fused after 2 days, and similar behavior was observed for all spheroids cryopreserved with the zwitterion/DMSO solutions and the commercial CPA.

5555 cancer cells in 5555/MAF1 co-cultured spheroids have the ability to invasion in collagen gels, similar to tumor tissues, with the aid of MAF1 cells^31^. We confirmed whether this distinctive ability was maintained post-thaw (Fig. 3b,c, Fig. S11). Invasion radii of 5555 cells after cryopreserving with ZD-5/5, ZD-5/10, ZD-5/15, and ZD-10/15 were 136, 120, 88, and 130 µm, respectively. These invasion radii were longer than that obtained with the commercial CPA (83 µm). MAF1 cells also have invasion ability and invasion radii of MAF1 cells were also observed just for information. Invasion radii after cryopreserving with ZD-5/5, ZD-5/10, ZD-5/15, and ZD-10/15 were 130, 126, 115, and 133 µm, respectively. These invasion radii were also longer than those obtained with the commercial CPA (95 µm). Although ZD-5/5 resulted in a similar cell recovery as the commercial CPA (Fig. 3a), it showed a higher invasion radius than that of the commercial CPA. Therefore, the zwitterion/DMSO aqueous solutions probably maintained the function of the co-cultured spheroids well.

### Cryopreservation of mouse tumor tissues

**Figure 4:**
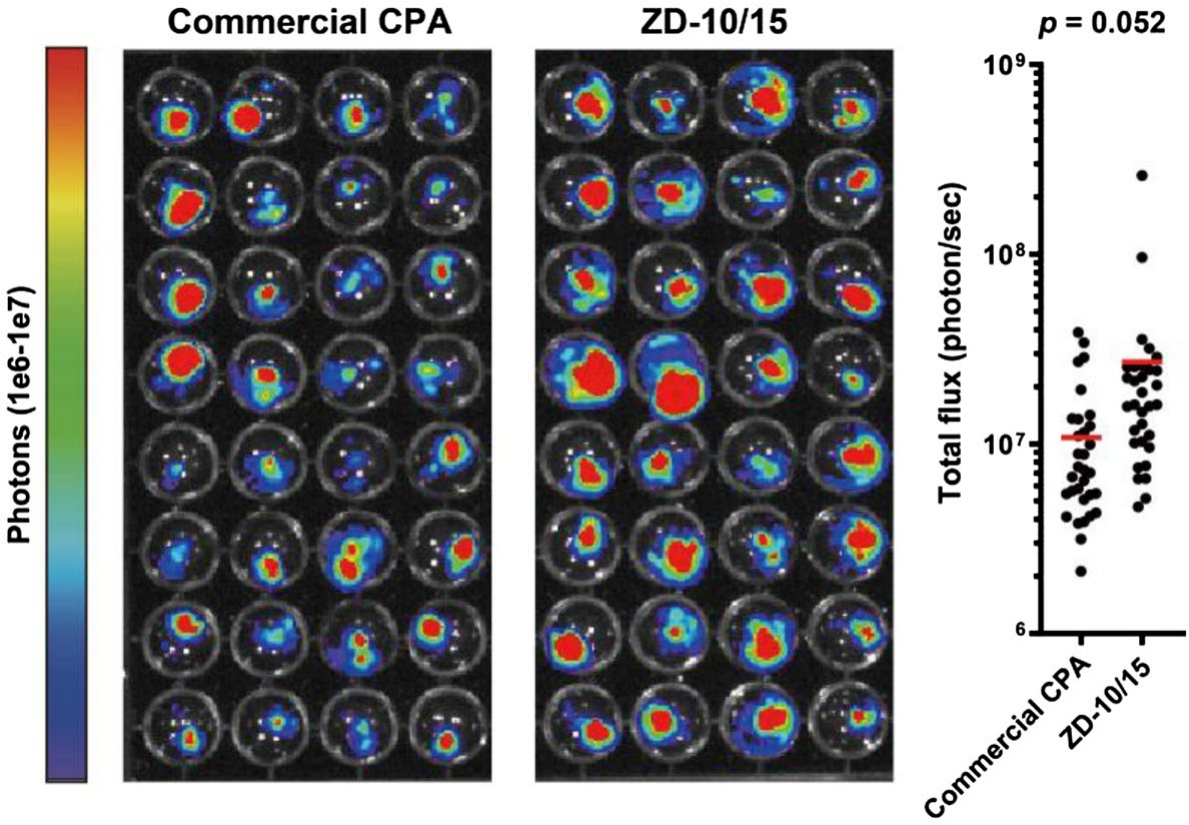
The relative number of living cells in cryopreserved mouse tumor tissues post-thaw using commercial CPA and ZD-10/15. Each tumor explant was quantified with IVIS and plotted. The commercial CPA employed was Cell Reservoir One (Nacalai Tesque).

We examined whether zwitterion/DMSO mixtures also support the cryopreservation of mouse tumor tissues as a different sample type having a more complex cellular composition than co-cultured spheroids. ZD-10/15, which showed the highest cryoprotective effect for 5555/MAF1 co-cultured spheroid, was therefore tested, in comparison to the commercially available CPA Cell Reservoir One (Nacalai Tesque). The number of surviving tumor cells was quantitatively evaluated by bioluminescent imaging. We found that tumor cells cryopreserved with ZD-10/15 exhibited a higher survival rate than those cryopreserved with the commercial CPA (Fig. 4). These results suggest that ZD-10/15 possesses a notable potential as a CPA not only for spheroids but also for more complex tumor tissues.

### Cryopreservation of human tumor tissues

**Figure 5:**
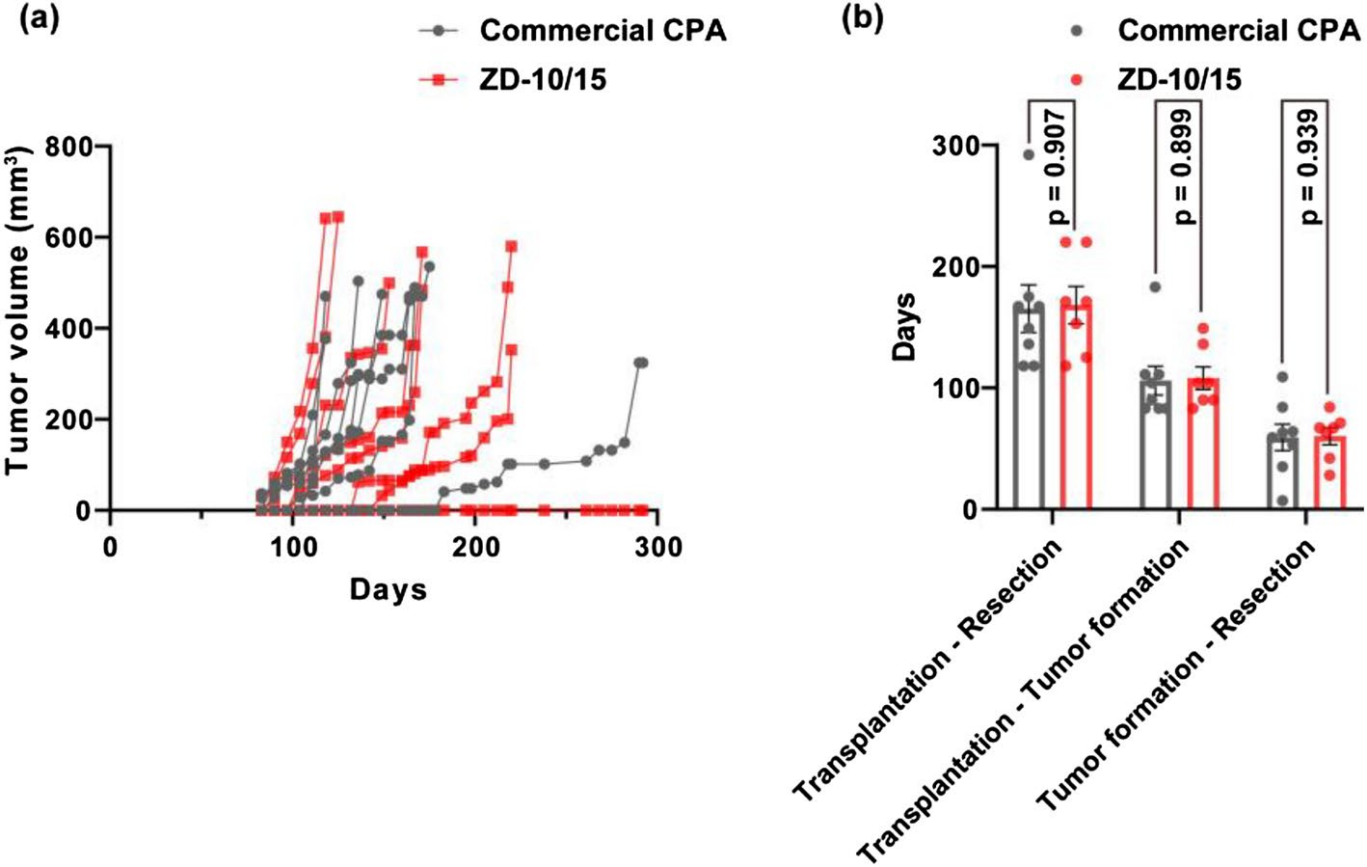
Tumor growth and its speeds of cryopreserved patient-derived xenografts using commercial CPA and ZD-10/15. (**a**) Tumor formation and growth of PDXs after cryopreservation with the commercial CPA or ZD-10/15. PDXs were resected at the final marked days. (**b**) The number of elapsed days from transplantation to resection (resection was carried out after growing by the long diameter of approximately 10 mm), from transplantation to tumor formation, and from tumor formation to resection. The bars show standard errors. The commercial CPA employed was Cell Reservoir One (Nacalai Tesque).

Transplantation of patient-derived breast cancer tissue xenografts (PDXs) into immunodeficient mice is an important technique in anticancer drug development at a preclinical stage, as the behavior of these tumors in mice was found to be similar to that of the tumor in its original recipient^32^. Therefore, next we cryopreserved PDXs using ZD-10/15 or a commercial CPA (Cell Reservoir One, Nacalai Tesque), thawed them, and transplanted them into mice. Out of eight PDXs that were thawed and transplanted into mice for both cryoprotection conditions, seven of those cryopreserved with ZD-10/15 engrafted and grew, versus eight in the commercial CPA group (Fig. 5a). The number of elapsed days from transplantation to resection (tumor resection was done after growing by the long diameter of approximately 10 mm), from transplantation to tumor formation, and from tumor formation to resection were shown in Fig. 5b. There were no significant differences between these number of elapsed days with ZD-10/15 and the commercial CPA (*p* value ≥ 0.899). ZD-10/15 did not show higher values than the commercial CPA in this PDX experiment whereas it showed higher values in the case of the mouse tumor tissues. However, ZD-10/15 exhibited cryoprotective effects comparable to those of the commercial CPA, which is practically used in the cryopreservation of PDXs. This indicates that ZD-10/15 can be immediately applied to the cryopreservation of human tissues such as PDXs.

## Conclusion

Mouse cell spheroids and mouse and human tumor tissues were cryopreserved by slow-freezing with the zwitterion aqueous solutions supplemented with DMSO. Following the cryopreservation of 5555, MAF1, and 5555/MAF1 spheroids, ZD-5/10, ZD-5/15, and ZD-10/15 produce higher post-thaw cell recoveries and invasion radii than those seen with the commercial CPA. ZD-10/15 exhibited the highest cryoprotective effect in 5555/MAF1 co-cultured spheroids. In mouse tumor tissues, using this cryoprotective solution also resulted in a higher number of living cells after thawing compared to a commercial CPA. With PDXs, ZD-10/15 was comparable to a commercial CPA. However, ZD-10/15 is a simple mixture of the zwitterion, DMSO, and water, and thus, there is significant room for further improvement in future works. For such improvement, the limited supply of non-commercial materials such as PDX would be a problematic issue.

## Materials and methods

### Materials

The zwitterion used in this study was synthesized as previously reported^29^. The reagents used to synthesize OE_2_imC_3_C (ethyl 4-bromobutyrate, imidazole, benzene sulfonyl chloride, and diethylene glycol monomethyl ether) were purchased from Tokyo Chemical Industry Co., Ltd., and used as received. The synthesis was confirmed by ^1^H nuclear magnetic resonance (NMR). Dimethyl sulfoxide (CultureSure^®^ DMSO) and CultureSure^®^ freezing medium were purchased from Fujifilm Wako Pure Chemical Corp. and used as received. Cell Reservoir One was purchased from Nacalai Tesque Inc.

### Cells

WM266.4 human melanoma cells, 5555 mouse melanoma cells, and MAF1 human melanoma-associated fibroblasts were kind gifts from Prof. Erik Sahai (Francis-Crick Institute) and were transduced with firefly luciferase and/or fluorescent proteins (mEGFP or mCherry) as described previously^31^.

### Cell culture

Cell culturing was conducted with Dulbecco’s modified Eagle’s medium (DMEM, high glucose with l-glutamine and phenol red, Fujifilm Wako Pure Chemical Corporation) supplemented with 1 vol% penicillin–streptomycin-amphotericin B suspension solution (Fujifilm Wako Pure Chemical Corporation) and 10 vol% FBS (Sigma-Aldrich Co., LLC) at 37 °C in 5% CO_2_ humidified atmosphere. The expanded cells were detached using trypsin solution (0.5 w/v% trypsin-5.3 mmol/L EDTA 4Na solution without phenol red, Fujifilm Wako Pure Chemical Corporation) for sub-culturing.

### Spheroid culture

Spheroids were prepared by the hanging-drop method. 2 × 10^6^ cells were collected and added into 2 mL medium supplemented with 0.6 w/v% methylcellulose (Sigma-Aldrich Co., LLC) in 2 mL sampling tubes; 20 µL of the cell suspension was placed on the inside of the culture plate’s covers. The covers were set on the culture plates filled with PBS and cells were incubated for 1 day at 37 °C in 5% CO_2_ humidified atmosphere.

### Cryopreservation of spheroids

Four spheroids were collected in 1.5-mL sampling tubes and washed with 1 mL PBS. After removing the supernatant, 100 µL CPA solution was slowly added at room temperature. The samples were immediately stored in a box (Mr. Frosty, Thermo Fisher Scientific Inc.) to cool at − 1 °C/min in a − 85 °C freezer for 1 week. The frozen samples were thawed with 1 mL medium (37 °C) and washed with 1 mL PBS for 3 min. After removing the supernatant, 50 µL trypsin solution was added and incubated at 37 °C for 30 min. Medium (50 µL) was added, and the cells were dispersed in the solution by pipetting. The cell suspension (10 µL) was mixed with 10 µL trypan blue (Fujifilm Wako Pure Chemical Corporation). The number of living cells and dead cells were counted using a hemocytometer (Fukaekasei Corporation and Watson Corporation). The cell recovery of spheroids was calculated with the following equation.

$$\text{Cell recovery }\left(\text{\%}\right)= \frac{\text{Counted living cell number }(\text{post-freeze})}{\text{Counted living cell number }(\text{pre-freeze})}\times 100$$

The relative cell recovery of spheroids was calculated with the following equation.

$$\text{Relative cell recovery}=\frac{\text{Cell recovery }(\text{sample freezing medium})}{\text{Cell recovery }(\text{commercial CPA})}$$

The relative cell recovery of 5555 spheroids after 24 h of incubation was calculated with the values after 24 h of incubation.

### Spheroid invasion

Cryopreserved 5555/MAF1 co-cultured spheroids were washed with 1 mL PBS for 3 min and transferred to glass bottom plates (Iwaki AGC Techno Glass Co., Ltd.) coated with 50 µL collagen gel. Collagen gel was made from Cellmatrix Type I-A (Nitta Gelatin Inc.), 1.72 × DMEM (made from DMEM powder, Thermo Fisher Scientific Inc.), and 1 M HEPES, and the mixing ratio was 4/5.8/0.2 in volume. After covering with 100 µL collagen, spheroids were incubated for 30 min, and 1 mL medium was added. After incubation for 24 h, images of the spheroids were captured with a microscope (ECLIPSE Ts2, Nikon Corporation). The radii of spheroids were calculated as true circles from the section area using the software “ImageJ” (ImageJ 1.52, Wayne Rasband, National Institutes of Health, USA). The invasion radii were calculated with the following equation.

$$\text{Invasion radius }\left(\mu\text{m}\right)=\text{Spheroid radius }\left(24\text{ hours}\right)-\text{Spheroid radius }(0\text{ hours})$$

### Cryopreservation of mouse tumors

WM266.4-Luc-mEGFP cells (2 × 10^6^ cells in 200 µL PBS (−)) were subcutaneously inoculated in both flanks of BALB/c slc nu/nu mice (purchased from SANKYO laboratory (Tokyo, Japan) and we used two mice for the experiment). After 25 days, the mice were euthanized and the subcutaneous tumors (< 1 cm in diameter) were excised, chopped into small pieces (approximately 1 mm^3^) and cryopreserved for 2 months (10–12 pieces in 500 µL CPA at − 80 °C). The tumor pieces were thawed and embedded into type I collagen gels (1.2 mg/mL) in a 96-well culture plate, covered with complete media and cultured for 24 h. Luciferin (300 µg/mL) was added into the wells and the number of viable WM266.4 cells in each tumor explant was quantified with a bioluminescent imaging system (IVIS Spectrum, PerkinElmer).

Animal experiments were done in accordance with the Institute for Experimental Animals of Kanazawa University (AP-184026).

### Cryopreservation of patient-derived xenografts

Breast cancer tissues obtained from breast cancer patients were cut into 1 mm squares, and three pieces were suspended in Matrigel^®^ (Corning) to make 50 µl of the mixture. Three pieces per site were inoculated into the mammary fat pads of immunodeficient non-obese diabetic (NOD)*-scid IL2rγ*^*null*^ (NSG) mice which were purchased from The Jackson Laboratory Japan, Inc. NSG mice were used in the animal experiments in this study to orthotopically implant patient-derived breast cancer tissue. This PDX mouse model is capable of reproducing human tumor tissues in mice. The purpose of the animal studies was to confirm the effectiveness of the PDX cryopreservation. Two mice were used for both control and experimental group. The quantification of functional cryopreserved PDX post-thaw was based on the number of abnormal mammary glands relative to the total number of inoculated mammary glands with a thawed PDX, that is, eight per experimental condition. No abnormalities were observed in all mice prior to animal experiments. Two hundred µL of three types of mixed anesthetic agents (0.3 mg/kg of medetomidine, 4 mg/kg of midazolam, and 5 mg/kg of butorphanol, medetomidine/midazolam/butorphanol = 0.3/4/5) was administered intraperitoneally per mouse, and the 3 pieces of patient-derived tumor tissue were implanted into the mammary gland using a trocar for mice. All the above procedures were performed in the animal room in the institute for experimental animals, Kanazawa University, Research Center for Experimental Modeling of Human Disease. Two hundred µL atipamezole (0.3 mg/kg) was administered immediately after the procedure. When tumors reached < 500 mm^3^, the tumors were resected. Tumor volume was measured using the following formula: V = L × L × S/2, where L and S are the lengths of the major axis and minor axis, respectively.

Human breast carcinoma specimens were obtained from the Kanazawa University Hospital. This study was approved by the institutional review boards of the Cancer Research Institute of Kanazawa University. Written informed consent was obtained from all participants before inclusion in the study. All experiments were conducted in accordance with the relevant guidelines and regulations. Animal experiments followed the recommendation of the ARRIVE guidelines, as described below. Mice were handled according to the guidelines of the Cancer Research Institute of Kanazawa University. The experiments were approved by committees for animal research at the Cancer Research Institute of Kanazawa University.

### Statistical analysis

Data were subjected to repeated measures of one-way ANOVA analysis, followed by Dunnett’s multiple comparison test. When two groups were compared, a two-tailed unpaired t-test was applied.

The software used for the tests is GraphPad Prism9. Experimental triplicates and quadruplicates in this study mean that the data were derived from independent experiments, but the cells collected from the same dishes were utilized.

## Supplementary Information

Supplementary Information.

## Data Availability

All data are available in the main text or the supplementary materials.
