# Supplementary material for: Cryopreservation of tissues by slow-freezing using an emerging zwitterionic cryoprotectant
Source: Sci Rep. 2023 Jan 2;13:37. doi: 10.1038/s41598-022-23913-3 (PMC9807565; doi:10.1038/s41598-022-23913-3)
Supplement: Supplementary file 1 — Supplementary Information. [file 41598_2022_23913_MOESM1_ESM.pdf]

Supporting Information

Cryopreservation of tissues by slow-freezing using an emerging zwitterionic cryoprotectant

Takeru Ishizaki, Kojiro Ishibashi, Yasuto Takeuchi, Noriko Gotoh, Eishu Hirata*, Kosuke Kuroda*

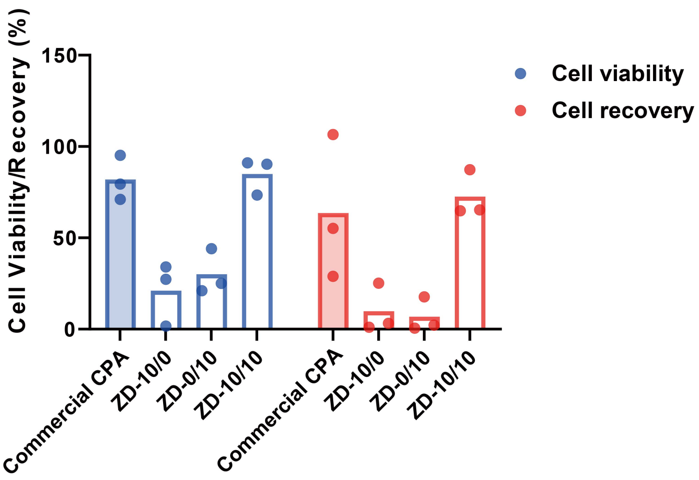

Fig. S1 Cell viabilities and recoveries of cryopreserved 5555 cell spheroids immediately post-thaw using non-optimized mixtures.

Cell viabilities and recoveries of 5555 cell spheroids after cryopreservation with the indicated solutions (*n*=1, experimental triplicates). The commercial cryoprotectant employed is CultureSure® freezing medium (Fujifilm Wako Pure Chemical Corporation).

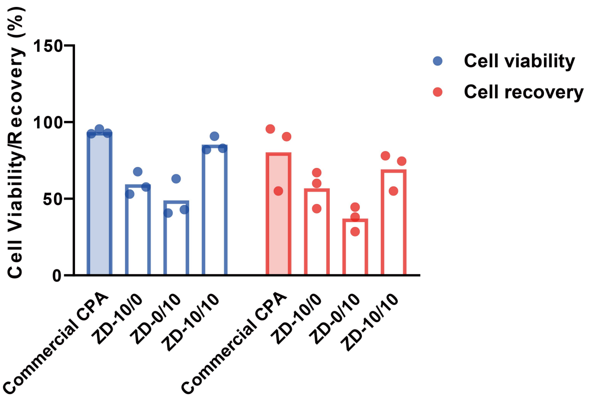

Fig. S2 Cell viabilities and recoveries of cryopreserved 5555 dispersed cells immediately post-thaw using non-optimized mixtures.

Cell viabilities and cell recoveries of 5555 dispersed cells after cryopreservation with the indicated solutions (*n*=1, experimental triplicates). The commercial cryoprotectant employed is CultureSure® freezing medium (Fujifilm Wako Pure Chemical Corporation).

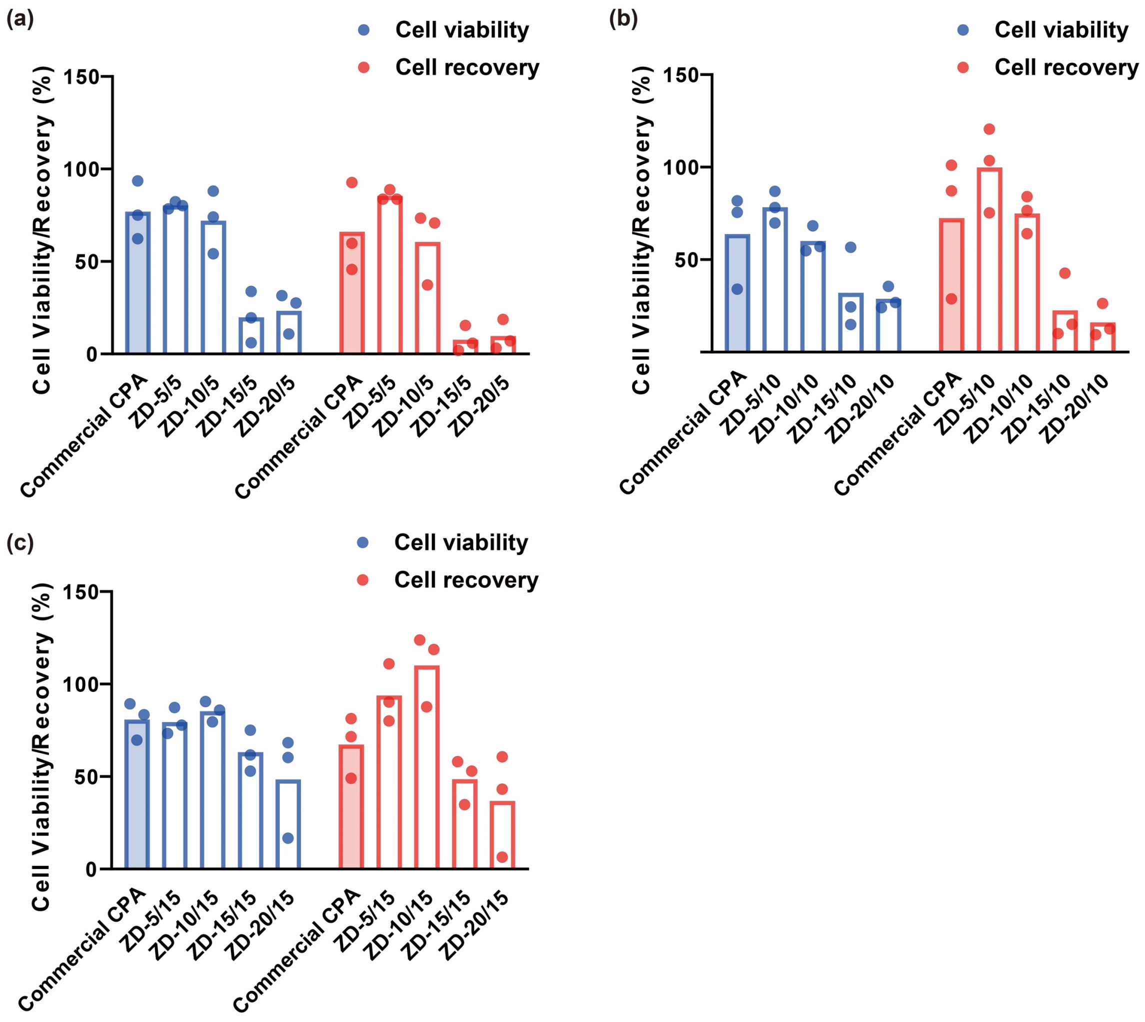

Fig. S3 Cell viabilities and recoveries of cryopreserved 5555 cell spheroids immediately post-thaw using different mixtures of zwitterion and DMSO.

Cell viabilities and cell recoveries of 5555 cell spheroids after cryopreservation with the indicated solutions (*n*=1, experimental triplicates). The commercial cryoprotectant employed is CultureSure® freezing medium (Fujifilm Wako Pure Chemical Corporation).

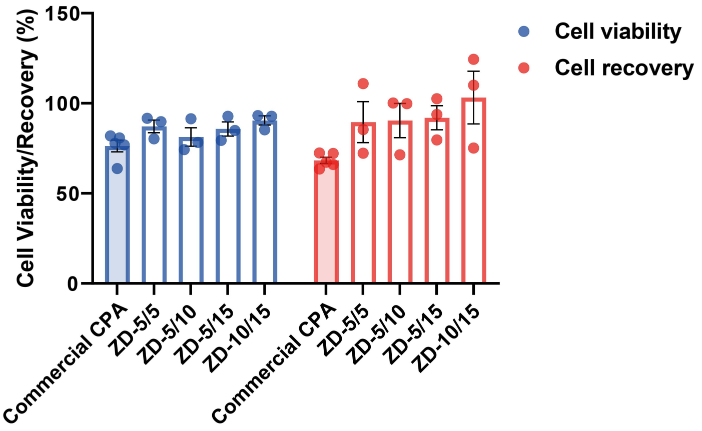

Fig. S4 Cell viabilities and recoveries of cryopreserved 5555 cell spheroids immediately post-thaw using optimized mixtures.

Cell viabilities and recoveries of 5555 cell spheroids after cryopreservation with the indicated solutions (n=3, biological triplicates). The bars show standard error. The commercial cryoprotectant employed is CultureSure® freezing medium (Fujifilm Wako Pure Chemical Corporation).

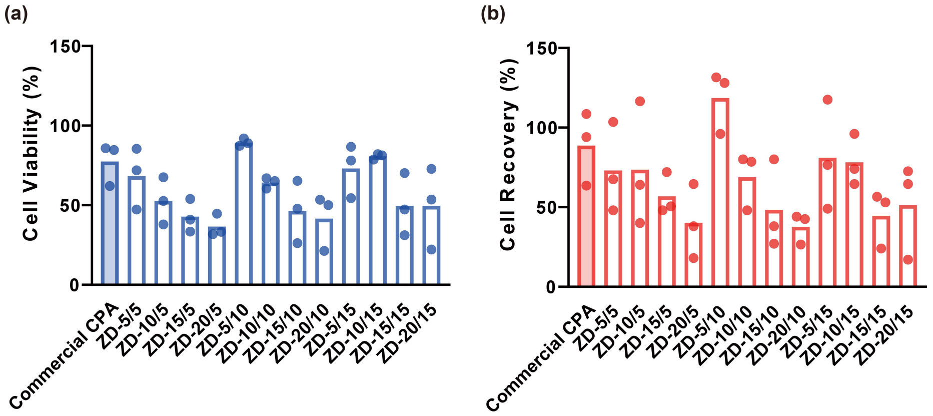

Fig. S5 Cell viabilities and recoveries of cryopreserved 5555 dispersed cells immediately post-thaw using different mixtures of zwitterion and DMSO.

Cell viabilities and recoveries of 5555 dispersed cells after cryopreservation with the indicated solutions (*n*=1, experimental triplicates). The commercial cryoprotectant employed is CultureSure® freezing medium (Fujifilm Wako Pure Chemical Corporation).

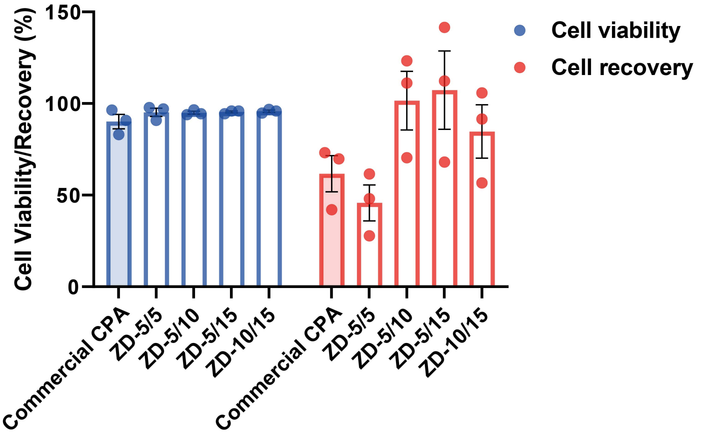

Fig. S6 Cell viabilities and recoveries of cryopreserved 5555 cell spheroids measured after 24 hours of incubation post-thaw using optimized mixtures.

Cell viabilities and recoveries of 5555 cell spheroids cryopreserved with the indicated solutions after 24 hours incubation (*n*=3, biological triplicates). The bars show standard error. The commercial cryoprotectant employed is CultureSure® freezing medium (Fujifilm Wako Pure Chemical Corporation).

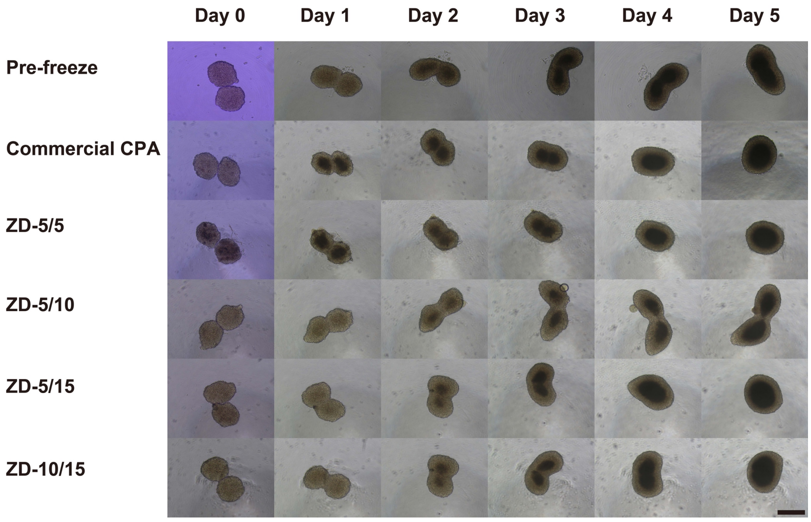

Fig. S7 Fusion activities of the cryopreserved 5555 cell spheroids post-thaw.

Microscopic observation of two spheroids cryopreserved with the indicated solution in a round-bottom non-adhesive dish for 5 days. Scale bar represents 500µm.

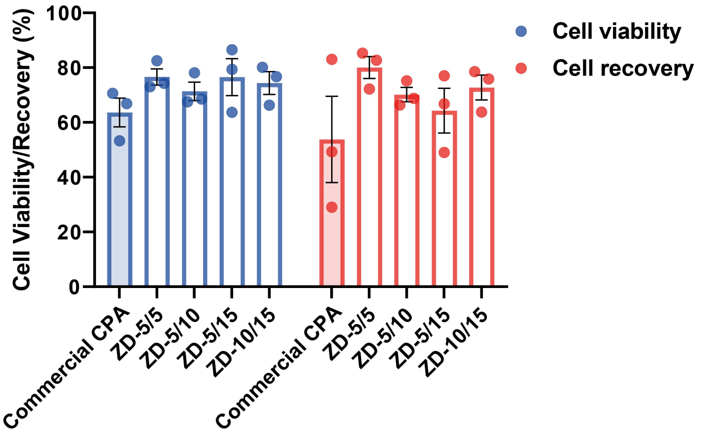

Fig. S8 Cell viabilities and recoveries of cryopreserved MAF1 cell spheroids immediately post-thaw using optimized mixtures.

Cell viabilities and recoveries of MAF1 cell spheroids after cryopreservation with the indicated solutions (*n*=3, biological triplicates). The bars show standard error. The commercial cryoprotectant employed is CultureSure® freezing medium (Fujifilm Wako Pure Chemical Corporation).

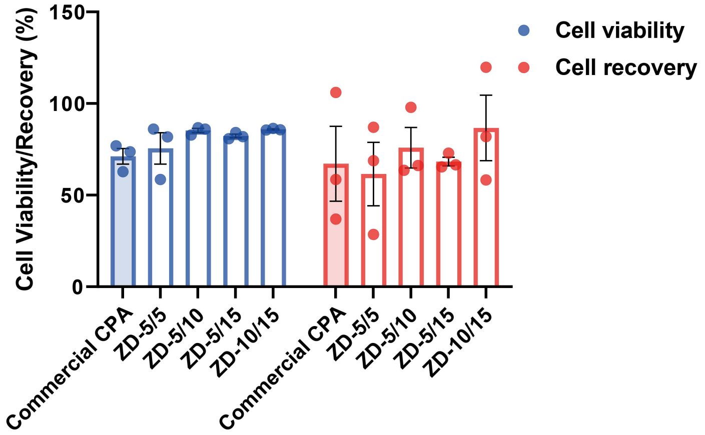

Fig. S9 Cell viabilities and recoveries of cryopreserved 5555/MAF1 cell spheroids immediately post-thaw using optimized mixtures.

Cell viabilities and recoveries of 5555/MAF1 cell co-cultured spheroids after cryopreservation with the indicated solutions (*n*=3, biological triplicates). The bars show standard error. The commercial cryoprotectant employed is CultureSure® freezing medium (Fujifilm Wako Pure Chemical Corporation).

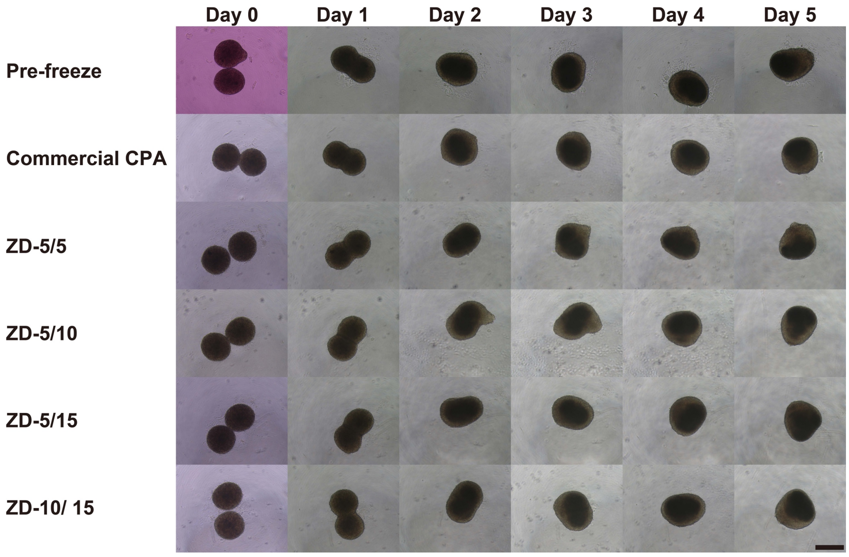

Fig. S10 Fusion activities of the cryopreserved 5555/MAF1 cell co-cultured spheroids post-thaw.

Microscopic observation of two spheroids cryopreserved with the indicated solution in a round-bottom non-adhesive dish for 5 days. Scale bar represents 500μm.

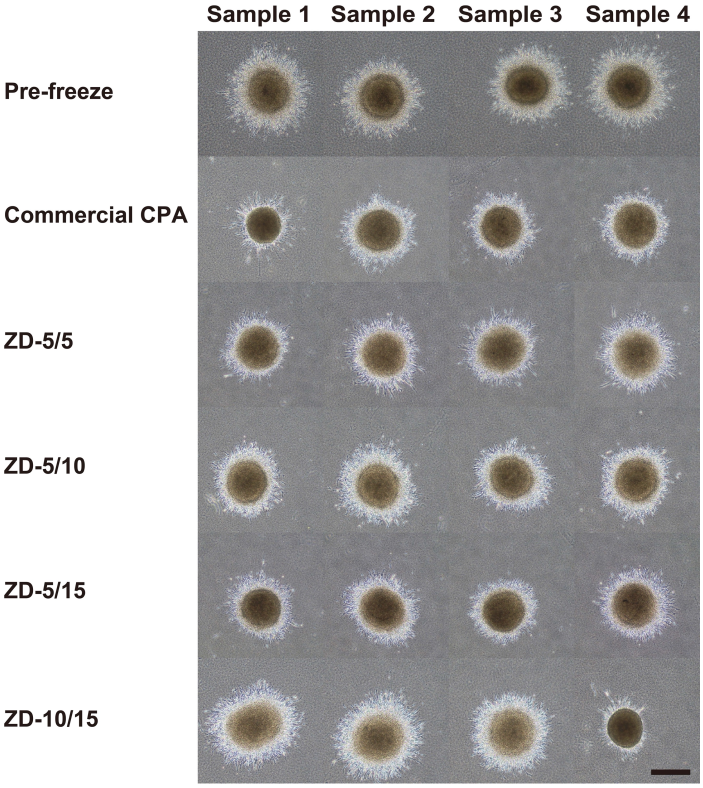

Fig. S11 Invasion ability of 5555/MAF1 cell co-cultured spheroids

Invasion of 5555/MAF1 cell co-cultured spheroids after 24h incubation in collagen gel. Scale bar represents 500 μm.

SUPPLEMENTAL EXPERIMENTAL PROCEDURES

Cell Cryopreservation

1×10^6 cells were collected in 1.5 mL sampling tubes and centrifuged (100 G, 5 min at room temperature). After removing the supernatant, cells were suspended slowly with 100 µL cryoprotectant solutions. The samples were stored in a box (Mr. Frosty, Thermo Fisher Scientific Inc.) to cool at -1 °C /min in a –85 °C freezer for one week. The frozen samples were thawed by 1 mL medium (37 °C) and centrifuged (100 G, 5 min at room temperature). After removing supernatant, cells were suspended with 1 mL medium. The cell suspension (10 µL) was mixed with 10 µL trypan blue (Fujifilm Wako Pure Chemical Corporation). The number of living cells and dead cells were counted using hemocytometer (Fukaekasei Corporation and Watson Corporation). The cell viability and cell recovery were calculated as following equations.

$$\text{Cell viability (\%)} = \frac{\text{Number of living cells}}{\text{Number of living cells} + \text{Number of dead cells}} \times 100$$

$$\text{Cell recovery (\%)} = \frac{\text{Number of living cells (post– freeze)}}{\text{Number of living cells (pre– freeze)}} \times 100$$

Cell viability after cryopreservation of spheroid

The cell viability of spheroids was calculated as following equations.

$$\text{Cell viability (\%)} = \frac{\text{Number of living cells}}{\text{Number of living cells} + \text{Number of dead cells}} \times 100$$

Fusion ability of spheroids

5555 and 5555/MAF1 co-cultured spheroids after cryopreservation were washed with PBS for 3 min. Two spheroids were put in a 96-well round-bottom non-adhesive plate (Sumitomo Bakelite Co., LTD.) with 100µL medium. Images of the spheroids for each day were captured with a microscope (ECLIPSE Ts2, Nikon Corporation).
